# Supplementary material for: Responses of microbial community from tropical pristine coastal soil to crude oil contamination
Source: PeerJ. 2016 Feb 18;4:e1733. doi: 10.7717/peerj.1733 (PMC4768689; doi:10.7717/peerj.1733)
Supplement: Table S1 [file peerj-04-1733-s001.docx]

**Supplementary tables**

**Table S1** – Number of quality filtered Illumina® barcoded sequences

| **Treatments** | **16S rRNA gene** | **Fungal Intergenic Spacer (ITS1)** |
| --- | --- | --- |
| Crude Oil | 45690 | 25315 |
| Crude Oil | 47917 | 46921 |
| Crude Oil | 52889 | 55579 |
| Control | 54458 | 91866 |
| Control | 55487 | 100736 |
| Control | 58337 | 103852 |
| Total | 314748 | 424269 |
